# Ageing-associated changes in the expression of lncRNAs in human tissues reflect a transcriptional modulation in ageing pathways

**DOI:** 10.1016/j.mad.2019.111177

**Published:** 2020-01

**Authors:** Saara Marttila, Kasit Chatsirisupachai, Daniel Palmer, João Pedro de Magalhães

**Affiliations:** Integrative Genomics of Ageing Group, Institute of Ageing and Chronic Disease, University of Liverpool, William Henry Duncan Building, 6 West Derby Street, Liverpool, L7 8TX, UK

**Keywords:** Aging, Transcriptomics, lncRNA, ncRNA

## Abstract

Ageing-associated changes in the protein coding transcriptome have been extensively characterised, but less attention has been paid to the non-coding portion of the human genome, especially to long non-coding RNAs (lncRNAs). Only a minority of known lncRNAs have been functionally characterised; however, a handful of these lncRNAs have already been linked to ageing-associated processes. To gain more information on the effects of ageing on lncRNAs, we identified from GTEx data lncRNAs that show ageing-associated expression patterns (age-lncRNAs) in 29 human tissues in 20-79-year-old individuals.

The age-lncRNAs identified were highly tissue-specific, but the protein coding genes co-expressed with the age-lncRNAs and the functional categories associated with the age-lncRNAs showed significant overlap across tissues. Functions associated with the age-lncRNAs, including immune system processes and transcription, were similar to what has previously been reported for protein coding genes with ageing-associated expression pattern. As the tissue-specific age-lncRNAs were associated with shared functions across tissues, they may reflect the tissue-specific fine-tuning of the common ageing-associated processes. The present study can be utilised as a resource when selecting and prioritising lncRNAs for further functional analyses.

## Highlights

• Ageing-associated changes in the expression of lncRNAs are highly tissue-specific.
• Ageing-associated lncRNAs are associated with similar functions across tissues.
• Ageing-associated changes in lncRNAs mirror those observed for protein coding genes.

## 1 Introduction

Despite the small proportion of protein coding sequences (3 %) in the human genome, the great majority of the genome is pervasively transcribed, producing a versatile pool of non-coding RNA molecules (ncRNAs) (ENCODE, 2012; Derrien et al., 2012; Djebali et al., 2012; Iyer et al., 2015). ncRNAs over 200 nt in length are classified as long non-coding RNAs (lncRNAs), and their number is at least comparable to that of protein coding genes and may be as high as 200 000 (Iyer et al., 2015; Xu et al., 2017; Hon et al., 2017). The processing of lncRNA molecules shares majority of features with mRNAs (Samudyata and Bonetti, 2018; Jarroux et al., 2017), but lncRNAs are typically expressed at a lower level as compared to protein coding genes. lncRNAs are very tissue- or lineage-specific and typically show highly specific spatio-temporal expression patterns (Djebali et al., 2012; Cabili et al., 2011; Ward et al., 2015; Li et al., 2015; Hon et al., 2017).

As everything over 200 nt in length and without protein coding potential is classified as a lncRNA, lncRNAs are a very heterogenous group. Only a minority of the identified lncRNAs have been functionally characterised, but the ones that have been characterised have been shown for example to regulate gene expression, post-transcriptional maturation, translation and epigenetics. Mechanisms by which lncRNAs bring about their function include interaction with other RNA species or DNA, scaffolding of subcellular domains or complexes and regulation of protein activity or abundance. In addition to the lncRNA transcript itself being functional, there is evidence showing that the act of transcription from the lncRNA locus can affect nuclear structure, epigenetic landscape or the expression of nearby genes (Ulitsky and Bartel, 2013; Kaikkonen and Adelman, 2018; Schmitz et al., 2016; Cech and Steitz, 2014; Yang et al., 2014; Melé and Rinn, 2016). lncRNAs have been shown to play a role for example in cellular pluripotency, cell differentiation, lineage specification, maintenance of cell identity, developmental patterning, dosage compensation and imprinting as well as cell migration (Flynn and Chang, 2014; Ransohoff et al., 2018).

Previously lncRNAs have been shown to be associated with processes important for various ageing-associated diseases, including cancer (He et al., 2019; Kondo et al., 2017), cardiovascular diseases (Bink et al., 2019; Zhou et al., 2016), type II diabetes (He et al., 2017) and neurodegenerative diseases such as Alzheimers disease (Pereira Fernandes et al., 2018; Idda et al., 2018). In model organisms, changes in the expression of lncRNAs with ageing have been reported (Wood et al., 2013). In humans, ageing-associated changes in the protein coding transcriptome have been extensively studied (Gomez-Verjan et al., 2018; Frenk and Houseley, 2018), but the ageing-associated changes in the non-coding transcriptome remain to be extensively studied. In the present study, we characterised the ageing-associated changes in lncRNA expression in 29 healthy human tissues between the ages 20 and 79 years, using the data available in GTEx portal (2019).

## 2 Materials & methods

### 2.1 Identifying ageing-associated lncRNAs

The data used for the analyses described in this manuscript were obtained from GTEx project, dbGaP accession number phs000424.v7. The GTEx data set consists of tissue-specific gene expression data from non-diseased tissues (gtexportal.org). Only samples from subject who died in a ventilator (Hardy scale 0) were included. We decided to restrict our analysis to subjects with the same death circumstance, as we found that death circumstance has an effect on gene expression profiles that could obscure true ageing-associated effects (described in more detail in Supplementary file 1). The sample number for each tissue studied varied from 6 to 733 (median 154) (Table 1).

**Table 1:** Details of sample counts, total number of expressed genes and lncRNAs as well as numbers of identified ageing associated genes and age-lncRNAs. Tissues with >10 age-lncRNAs were included in further analyses (shown here in bold).

Tissue (number of samples)	Total expressed	Expressed lncRNAs	Total ageing-associated	Age-lncRNAs (percentage of expressed lncRNAs)	Up/down-regulated age-lncRNAs
**Adipose (457)**	**17299**	**3531**	**279**	**35 (1.0)**	**13/22**
**Adrenal (157)**	**16009**	**2893**	**374**	**37 (1.3)**	**26/11**
Bladder (11)	18059	3986	0	0 (0)	0/0
Blood (325)	14349	2431	0	0 (0)	0/0
**Blood vessel (549)**	**17031**	**3558**	**1578**	**205 (5.8)**	**164/41**
**Brain (69)**	**17154**	**3309**	**336**	**112 (3.4)**	**58/54**
Breast (168)	18159	3992	19	2 (0.05)	2/0
**Cervix uteri (11)**	**18843**	**4385**	**1882**	**189 (4.3)**	**100/89**
**Colon (362)**	**17642**	**3511**	**285**	**45 (1.3)**	**19/26**
**Esophagus (733)**	**17371**	**3374**	**99**	**20 (0.6)**	**14/6**
**Heart (330)**	**14286**	**1946**	**281**	**53 (2.7)**	**24/29**
Kidney (15)	16927	3238	0	0 (0)	0/0
Liver (69)	14181	2213	31	3 (0.1)	2/1
**Lung (241)**	**18820**	**4258**	**1182**	**164 (3.9)**	**111/53**
**Muscle (300)**	**13488**	**1915**	**391**	**62 (3.2)**	**33/29**
**Nerve (224)**	**18602**	**4611**	**505**	**52 (1.1)**	**26/26**
**Ovary (91)**	**18258**	**4613**	**309**	**29 (0.6)**	**11/18**
Pancreas (214)	14531	2183	23	3 (0.1)	2/1
Pituitary (6)	19170	4648	0	0 (0)	0/0
**Prostate (101)**	**19058**	**4572**	**2042**	**346 (7.6)**	**77/269**
**Salivary (67)**	**17711**	**3585**	**490**	**40 (1.1)**	**12/28**
Skin (635)	17631	3608	5	0 (0)	0/0
Small intestine (127)	18254	3851	0	0 (0)	0/0
Spleen (151)	18182	4332	26	7 (0.2)	6/1
**Stomach (226)**	**16894**	**3081**	**82**	**15 (0.5)**	**10/5**
Testis (148)	25213	9254	22	7 (0.08)	4/3
Thyroid (253)	18995	4795	25	5 (0.1)	0/5
**Uterus (79)**	**18630**	**4546**	**847**	**101 (2.2)**	**60/41**
Vagina (80)	18677	4274	1	0 (0)	0/0

For each tissue, we identified differentially expressed genes with age using the following linear regression model:

Y_ij_ = αAge_i_ + βSex_i_ +ε_ij_

where *Y_ij_* is the expression level of gene *j* in sample *i*, *Age_i_* denotes the age of sample *i, Sex_i_* denotes the sex of sample *i* and *ε_ij_* denotes the error term. The dataset provided the age of each subject as an age range (20–29, 30–39, 40–49, 50–59, 60–69 and 70–79); we approximated the age of each sample to be 25, 35, 45, 55, 65 and 75, respectively. To remove genes with low expression values, we excluded genes with expression less than 1 count per million (cpm) in more than 30 percent of samples. Raw read counts were normalized using TMM normalization and were voom transformed to remove heteroscedasticity from the count data. The linear model for each gene was generated by using the limma package in R (version 3.36.5). Genes were considered significantly associated with age with empirical Bayes moderated t-statistics and their associated adjust P-value (Benjamini-Hochberg method) < 0.05 and absolute fold change across 50 years (from 25 to 75 years old) > 1.5.

Biotypes of the ageing-associated genes were identified using the R package biomaRt (Durinck et al., 2009), based on Ensembl release 92 (April 2018). Protein coding genes as well as immunoglobulin genes were removed from the results, and the remaining non-coding genes were used in the following analyses. The total number of ageing-associated genes ranged from 0 to 2042 across tissues (0–10.8 % of all expressed genes) and the number of age-lncRNAs ranged from 0 to 346 (0–33.3 % of all ageing associated genes), for each tissue the numbers of ageing associated genes and ageing-associated lncRNAs (age-lncRNAs) are shown in Table 1.

### 2.2 Tissue specificity index (Tau)

For all genes in the GTEx data, a τ tissue speciﬁcity index was calculated. The τ index is an indicator of how speciﬁcally or broadly expressed a gene is, with a τ of 1 indicating expression speciﬁc to only one tissue, and a τ of 0 indicating equal expression across all tissues (Yanai et al., 2005). The τ index for a given gene can be calculated using the following equation:

$$\tau =\frac{\sum_{i-1}^{N}\left(1-x_{i}\right)}{N-1}$$

where *N* is the number of tissues being studied and *x_i_* is the expression proﬁle component for a given tissue, normalised by the maximal component value for that gene (i.e. the expression of that gene in the tissue it is most highly expressed in).

### 2.3 Genes co-expressed with age-lncRNAs

Genes co-expressed with age-lncRNAs were identified with GeneFriends RNA-seq (v3.1) (van Dam et al., 2015). This co-expression analysis describes which genes tend to be activated along with the age-lncRNAs of interest, which can be thus assumed to be under similar transcriptional regulation and to participate in similar functions. It does not, however, suggest regulatory relationship between the lncRNAs themselves and genes co-expressed with them. For each tissue, and separately for up- and down-regulated, age-lncRNAs were used as input (for numbers of age-lncRNAs, see Table 1) (data downloaded on 12.3.2019). The resulting list of co-expressed genes was trimmed to include only genes expressed in the tissue in question in GTEx data (median expression TPM > 1). The top 5 % of the co-expressed genes were used in further analyses.

The overlap of lists of co-expressed genes was analysed using the R package OrderedLists (Yang et al., 2018), which yields a similarity score of the two lists, giving emphasis to genes in the top ranks. For the comparisons, an empirical p-value is calculated based on random shuffling of the original list. Tissues were compared to each other pairwise, with two.sided set to “FALSE” in order to only compare the top members of the list. 10,000 permutations were performed to estimate empirical p-values, with p-values < 0.05 considered significant.

### 2.4 Functional enrichment and semantic similarity of GO terms

Enrichment of GO terms was analysed with R package topGO (Alexa and Rahnenfuhrer, 2018), which takes into account the hierarchical structure of GO terms. For each tissue, the enrichment was analysed for the genes co-expressed with the age-lncRNAs using the “weight01” method and using BH-corrected p-value of 0.05 as a cut-off.

The semantic similarity of lists of enriched GO terms between tissues was analysed with G-SESAME (James et al., 2007). This method takes into account the ancestors of each GO term. For the analysis, default settings were used, semantic contribution factors for “is_a” and “part_of” relationships were set to 0.8 and 0.6, respectively. Sematic similarity was calculated for all tissue pairs, separately for up- and down-regulated, and excluding tissues with no or only one statistically significant GO term. Lists of GO terms were considered to be similar with similarity score >0.5.

Enrichment of KEGG terms was analysed with FunnMapOne (Scala et al., 2019), which was also used to visualise the enrichment of KEGG terms. For the enrichment, terms with p-value < 0.05 were considered significant. For the visualisation and clustering of tissues, default settings of FunMappOne were used.

## 3 Results

### 3.1 Ageing-associated changes in the expression of lncRNAs

Across 29 analysed tissues, we identified lncRNAs with ageing-associated expression patterns in 22 tissues. The number of lncRNAs with an ageing-associated expression pattern (age-lncRNAs) varied from 2 to 246 per tissue (median 38.5, 0.05–7.6 % of all expressed lncRNAs, see Table 1). In total there were 612 age-lncRNAs up-regulated and 652 age-lncRNAs down-regulated across tissues. Of the up-regulated age-lncRNAs, 29 showed an ageing-associated expression pattern in three or more tissues and the great majority, 490 age-lncRNAs, were ageing-associated in only one tissue. For down-regulated, these numbers are 16 and 570, respectively. Age-lncRNAs, that showed an association with ageing in three or more tissues are hereafter referred as multi-tissue age-lncRNAs. In addition, there were 112 age-lncRNAs that showed a varied expression with ageing and that were up-regulated in one or more tissues and down-regulated in another tissue or vice versa. All age-lncRNAs are presented in Supplementary Table 1. There were 16 tissues with 10 or more age-lncRNAs, and these tissues are used in further analyses (adipose tissue, adrenal gland, blood vessel, brain, cervix uteri, colon, esophagus, heart, lung, muscle, nerve, ovary, prostate, salivary gland, stomach and uterus).

We compared the identified age-lncRNAs to lncRNAs previously reported to be ageing associated in individual tissues, in peripheral blood mononuclear cells, (PBMCs, Noren Hooten and Evans, 2019), tendon (Peffers et al., 2015) and brain subependymal zone (SEZ, Barry et al., 2015). GTEx does not contain expression data from PBMCs or tendon and comparing the ageing-associated lncRNAs identified in these tissues to all age-lncRNAs identified in the present study revealed only a modest overlap. There were 1938 ageing-associated lncRNAs in PBMCs, of which 117 were identified in the present study and 62 lncRNAs in tendon, of which 12 were identified in the present study. While GTEx contains several brain regions, SEZ is not included. Of the 6 ageing-associated lncRNAs reported in SEZ, none are age-lncRNAs in the brain, but 3 were identified as age-lncRNAs in other tissues.

lncRNAs can be characterised as ageing-associated based on their known molecular function and involvement of this function with the ageing process. Three reviews have compiled lists of such lncRNAs (Grammatikakis et al., 2014; Costa et al., 2016; Kour and Rath, 2016). In our study, 9 lncRNAs characterised as ageing-associated in these reviews were identified as age-lncRNAs (Table 2). In addition, expression of lncRNAs in cellular senescence has been characterised (Abdelmohsen et al., 2013), and 83 lncRNAs were reported to be differentially expressed between proliferating and senescent cells. Of these 16 were identified as age-lncRNAs in the present study (Table 3). As shown in Table 2, Table 3, lncRNAs previously reported to be associated with either ageing or senescence showed both up- and down-regulation with age in different human tissues.

**Table 2:** lncRNAs previously classified as ageing-associated that were identified as age-lncRNAs in the present study.

lncRNA		Function	Present study	Reviewed in
XIST	ENSG00000229807	Imprinting and silencing of X chromosome, down-regulated in senescence	Brain (down)	Grammatikakis et al., 2014; Kour and Rath, 2016
H19	ENSG00000130600	Imprinting of H19/IGF2 locus, involved in cell growth and proliferation, development and growth. Up-regulated with ageing	Muscle (up)	Grammatikakis et al., 2014; Kour and Rath, 2016
TARID	ENSG00000227954	Induction of TCF21 expression by promoter demethylation	Lung (up)	Grammatikakis et al., 2014; Costa et al., 2015
MEG3	ENSG00000214548	A tumor suppressor that interacts with p53, levels inversely correlate with autophagy levels	Cervix uteri (up); Lung (up)	Grammatikakis et al., 2014; Kour and Rath, 2016
HOTAIR	ENSG00000228630	Involved in development and imprinting, up-regulated with senescence	Blood vessel (down)	Grammatikakis et al., 2014; Costa et al., 2015; Kour &Rath, 2016
MALAT1	ENSG00000251562	Needed for cell proliferation, down-regulated in senescence	Uterus (up)	Grammatikakis et al., 2014; Costa et al., 2015; Kour and Rath, 2016
CRNDE	ENSG00000245694	Regulated by insulin/IGF, involved in regulation of cellular metabolism	Lung (down); uterus (up)	Costa et al., 2015
NEAT1	ENSG00000245532	Involved in nuclear formation	Prostate (down)	Costa et al., 2015; Kour and Rath, 2016
MIAT	ENSG00000225783	Decreased in senescent cells	Blood vessel (up); Cervix uteri (down)	Kour and Rath, 2016

**Table 3:** lncRNAs identified to be differentially expressed between senescent and proliferating cells, that were also identified as age-lncRNAs in the present study.

age-lncRNA		Senescence (Abdelmohsen et al., 2013)	Present study
ENSG00000230918	AC008063.2	Up	Prostate (down)
ENSG00000237424	FOXD2-AS1	Down	Esophagus (up), Prostate (up)
ENSG00000196205	EEF1A1P5	Down	Brain (down), Lung (down), Muscle (up)
ENSG00000243742	RPLP0P2	Down	Prostate (down)
ENSG00000214826	DDX12P	Down	Blood vessel (up), Brain (down)
ENSG00000246596	AC139795.1	Down	Lung (up)
ENSG00000232274	BX571672.6	Up	Lung (up), Spleen (up)
ENSG00000238261	BX004987.5	Up	Lung (up)
ENSG00000235385	LINC02154	Up	Lung (up)
ENSG00000253864	AC131025.8	Down	Blood vessel (up), Cervix uteri (up), Lung (up), Prostate (up), Testis (up)
ENSG00000226562	CYP4F26P	Down	Prostate (down)
ENSG00000249669	CARMN, MIR143HG	Down	Cervix uteri (up), Lung (up), Prostate (up)
ENSG00000245532	NEAT1	Down	Prostate (down)
ENSG00000251562	MALAT1	Down	Uterus (up)
ENSG00000229807	XIST	Down	Brain (down)
ENSG00000225783	MIAT	Down	Blood vessel (up), Cervix uteri (down)

### 3.2 Tissue specificity of age-lncRNAs

Previously it has been reported that lncRNAs are more tissue specific as compared to protein coding genes (Djebali et al., 2012; Cabili et al., 2011; Ward et al., 2015; Li et al., 2015; Hon et al., 2017), and our results are in line with previous findings, as the protein coding genes expressed in the GTEx data had a lower Tau score as compared to the lncRNAs (0.36 and 0.66, respectively, Mann-Whitney *U* test p-value < 2.2e-16). When comparing all the up-regulated and down-regulated age-lncRNAs, we observed that the down-regulated age-lncRNAs are more tissue specific as compared to the up-regulated age-lncRNAs, although the absolute difference was modest (median Tau scores 0.75 and 0.65, respectively, Mann-Whitney *U* test p-value = 1.95e-08).

### 3.3 Protein coding genes co-expressed with age-lncRNAs

Very few lncRNAs are functionally characterised, so in order to gain insight to the possible functions of the identified age-lncRNAs, we utilised guilt-by-association analysis and identified the protein coding genes co-expressed with the age-lncRNAs. This was done for each tissue, separately for the up-and down-regulated age-lncRNAs as well as for the multi-tissue age-lncRNAs. The top 5 % of co-expressed genes for each tissue are presented in Supplementary Table 2.

**Fig. 1:**
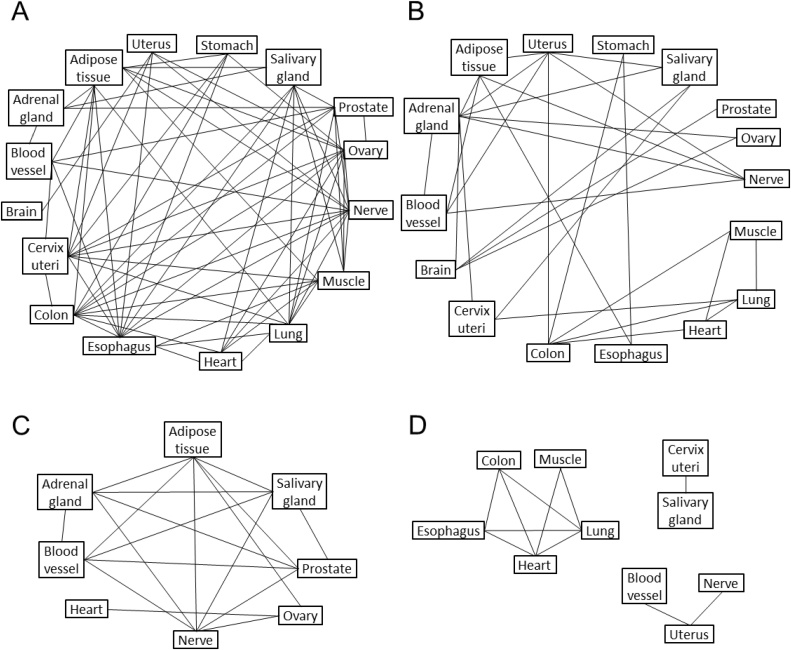
Overlap of the group of genes co-expressed with age-lncRNAs across tissues, for (A) up-regulated and (B) down-regulated age-lncRNAs. Tissues that had statistically significant overlap are connected with a line (see also Supplementary Table 3). Semantic similarity of GO terms between tissues, for (C) up-regulated and (D) down-regulated age-lncRNAs. Tissues with significant semantic similarity are connected with a line (see also Supplementary table 5). Tissues with less than 2 significant GO terms and tissues with no semantic similarity are not shown.

We analysed the overlap between lists of co-expressed genes in a pairwise manner for all tissues, separately for up- and down-regulated age-lncRNAs (Supplementary Table 3). Considering protein coding genes co-expressed with up-regulated age-lncRNAs, each tissue showed a statistically significant overlap with 1–12 other tissues (median 9.5) and for down-regulated, each tissue showed an overlap with 1–8 other tissues (median 4), the difference was statistically significant (Mann-Whitney *U* test p-value 0.000217) (Fig. 1), indicating that the up-regulated genes share more of the co-expressed genes across tissues as compared to down-regulated age-lncRNAs.

We then compared the genes co-expressed with multi-tissue age-lncRNAs to genes constituting a transcriptomic signature of ageing, obtained through a meta-analysis of ageing-associated transcriptomic changes (Palmer et al., 2019). This signature contains 449 genes commonly up-regulated with ageing and 162 genes commonly down-regulated with ageing. 82 of the up-regulated signature genes were co-expressed with up-regulated age-lncRNAs, which is more than expected by chance (hypergeometric test p-value 1.08e-26). However, there was no significant overlap between the down-regulated signature genes and genes co-expressed with down-regulated multi-tissue age-lncRNAs. We also compared the co-expressed genes to genes found in GenAge, the benchmark database of genes related to ageing (Tacutu et al., 2018). However, there was no statistically significant overlap between genes co-expressed with multi-tissue lncRNAs and genes in GenAge database.

### 3.4 Functional enrichment of co-expressed genes

We performed a functional enrichment analysis for the genes co-expressed with the age-lncRNAs. For the genes co-expressed with the up-regulated age-lncRNAs, we identified significantly enriched GO terms in 14 tissues and for the genes co-expressed with down-regulated age-lncRNAs, we identified significantly enriched GO terms in 13 tissues. All significant GO terms are presented in Supplementary Table 4. To get a quantitative view on the similarities between the enriched GO terms across tissues, we calculated the semantic similarity of the lists of GO terms. The semantic similarity was analysed pairwise across tissues, including tissues for which there were at least 2 enriched GO terms (Supplementary Table 5). For the up-regulated, there was significant semantic similarity for each tissue with 0–6 other tissues (median 2), for down-regulated 0–4 (median 1.5), the difference was not statistically significant. Fig. 1 shows the tissues which had significant semantic similarity between them.

GO terms associated with the up-regulated age-lncRNAs showed semantic similarity in adipose tissue, adrenal gland blood vessel, nerve, ovary, prostate and salivary gland (Supplementary Table 5, Fig. 1). In these tissues the majority of enriched GO terms were associated with immune system functions (Supplementary Table 4). Based on the semantic similarity of GO terms associated with down-regulated age-lncRNAs, we could identify three smaller clusters of tissues (Supplementary Table 5, Fig. 1). In colon, esophagus, heart, lung and muscle, the majority of enriched GO terms were also associated with immune system functions. In addition, GO terms in cervix uteri and salivary gland showed semantic similarity as well as GO terms in blood vessel, nerve and uterus (Fig. 1, Supplementary Table 4).

We also identified the enriched GO terms for the multi-tissue age-lncRNAs, that showed an ageing-associated expression pattern in 3 or more tissues. For the genes co-expressed with these up-regulated multi-tissue age-lncRNAs, a great majority of the enriched GO terms were immune system associated, in addition to GO terms associated with nucleotide metabolism, transcription, translation and protein homeostasis being enriched. For genes co-expressed with down-regulated multi-tissue age-lncRNAs, there was only one statistically significantly enriched GO term (Supplementary Table 4).

**Fig. 2:**
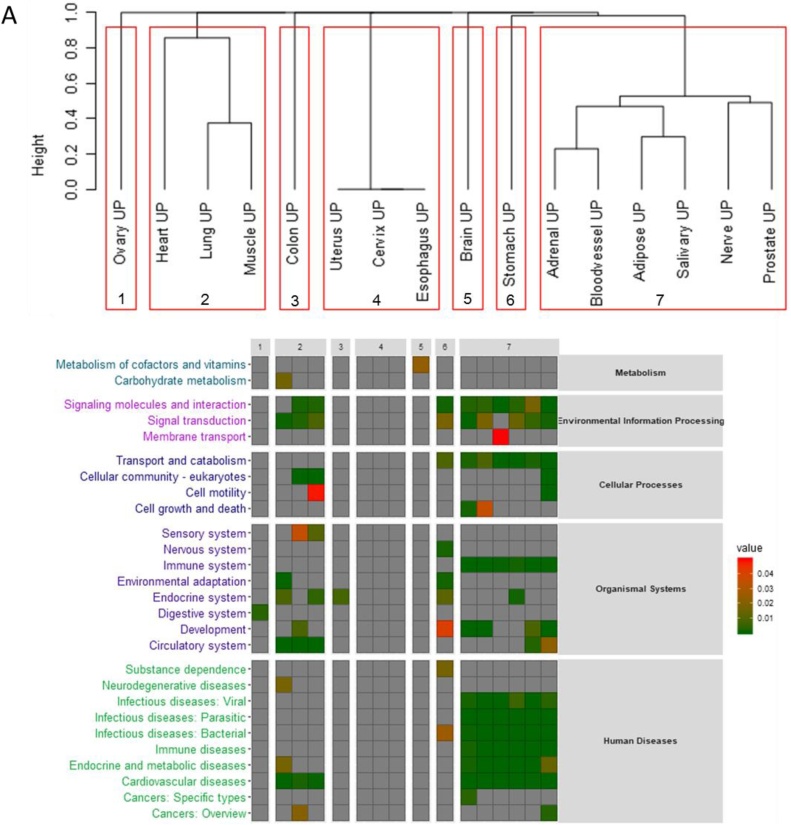

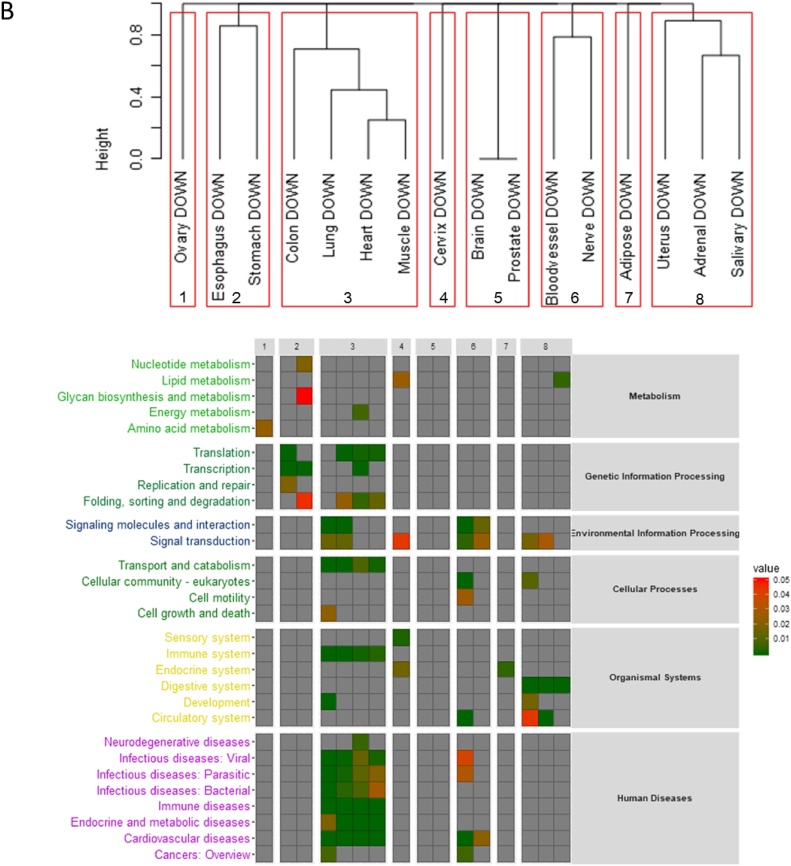
Enrichment of KEGG pathways for genes co-expressed with age-lncRNAs, in (A) for up-regulated and (B) for down-regulated. Tissues were clustered according to the enrichment of KEGG pathways, see Methods section for details.

Analysis of enrichment of KEGG pathways yielded similar results as the analysis of GO term enrichment. We identified statistically significant KEGG pathways in 13 tissues when analysing gene co-expressed with the up-regulated age-lncRNAs. Based on the enriched KEGG pathways, the tissues formed 2 clusters, with 4 tissues not clustering with any other (Fig. 2). The largest cluster consisted of adipose tissue, adrenal gland, blood vessel, nerve, prostate and salivary gland and these tissues showed an enrichment of immune system related KEGG terms, as well as those associated with signal transduction. Heart, lung and muscle formed another cluster, and showed enrichment of signal transduction, circulatory system and cardiovascular diseases.

For genes co-expressed with the down-regulated age-lncRNAs, we identified statistically significantly enriched KEGG terms for 14 tissues. Based on the enrichment of KEGG terms, these tissues formed 4 clusters, with 3 tissues not clustering with any other (Fig. 2). The largest cluster was formed by colon, lung, heart and muscle, and was enriched primarily for immune system processes. Other clusters were formed by esophagus and stomach, that showed enrichment in transcription and translation; uterus, adrenal gland and salivary gland that showed enrichment in digestive system process, and blood vessel and nerve, that showed enrichment in signalling molecules and signal transduction.

## 4 Discussion

### 4.1 Ageing-associated lncRNAs

We identified lncRNAs with an ageing-associated expression pattern in 22 healthy human tissues. Previous reports on transcriptomic changes with ageing have focused on protein coding genes, with little emphasis on the non-coding transcriptome (reviewed in Gomez-Verjan et al., 2018; Frenk and Houseley, 2018). A great majority of the identified age-lncRNAs were specific to only one tissue, and one lncRNA was identified to be ageing-associated in a maximum of 5 tissues. Comparing our results to those published by others (Noren Hooten and Evans, 2019; Peffers et al., 2015; Barry et al., 2015) also revealed minimal overlap between the identified age-lncRNAs, as did the comparison to lncRNAs defined as ageing-associated based on molecular function (Table 2) (Grammatikakis et al., 2014; Costa et al., 2016; Kour and Rath, 2016). These results are in line with previous results on ageing-associated changes in the protein coding transcriptome, where overlap between tissues and studies has been modest (reviewed in Gomez-Verjan et al., 2018; Frenk and Houseley, 2018). Our results and those reported previously do not suggest any single lncRNA as being associated with the ageing process in all or a majority of tissues.

### 4.2 Functions associated with age-lncRNAs

Despite being very tissue-specific, the age-lncRNAs identified shared co-expressed genes and were associated with a shared group of enriched GO and KEGG terms. Immune system function was associated with up-regulated lncRNAs in one group of tissues and down-regulated age-lncRNAs in another group of tissues (Fig. 2, Supplementary Table 4). Ageing-associated changes in the immune system associated protein coding genes have been widely reported previously (Irizar et al., 2015; Passtoors et al., 2012; Remondini et al., 2010; Reynolds et al., 2015; Erraji-Benchekroun et al., 2005; Rodwell et al., 2004; Zahn et al., 2006; Marttila et al., 2013; Harris et al., 2017; Muñoz-Culla et al., 2017), and dysregulation of immune system genes has been presented as one of the consensus pathways in ageing as well as proposed to be a gene expression hallmark of ageing (Cellerino and Ori, 2017; Frenk and Houseley, 2018; de Magalhães et al., 2009). On a larger scale, comprehensive dysregulation of immune system, with immunosenescence and inflamm-aging, is well described in the literature (Nikolich-Žugich, 2018; Fulop et al., 2018). It is interesting that both up-regulated and down-regulated age-lncRNAs are associated with immune system functions, depending on the tissue. Notably, tissues that are in contact with the external environment, colon, esophagus and lung all had down-regulated age-lncRNAs associated with immune system function. However, based on lncRNA expression changes alone, it is not possible to determine in which way the immune system is dysregulated in each tissue, for example, is inflamm-aging or immunosenescence more pronounced. In addition, changes in the number and proportions of tissue-residing immune cells may contribute to the observed changes in the expression of immune-system associated lncRNAs.

In addition to immune system dysregulation, downregulation of protein synthesis machinery and dysregulation of gene expression and mRNA processing have been proposed to be gene expression hallmarks of ageing (Frenk and Houseley, 2018). These processes were also identified to be associated with age-lncRNAs. Taken together, functional analysis of age-lncRNAs reveals similar functional categories as transcriptomic analyses performed on protein coding genes showing ageing-associated expression patterns (reviewed in Gomez-Verjan et al., 2018; Frenk and Houseley, 2018). Similarities of ageing-associated changes in lncRNA and protein coding gene expression were also suggested by the overlap of genes co-expressed with up-regulated multi-tissue age-lncRNAs and the transcriptomic signature of ageing (Palmer et al., 2019).

### 4.3 Tissue-specific age-lncRNAs with shared co-expression partners and functions

The identified age-lncRNAs were highly tissue-specific, and only little overlap was observed with lncRNAs previously identified as ageing-associate in other tissues (Noren Hooten and Evans, 2019; Peffers et al., 2015; Barry et al., 2015). This is not surprising, given the know tissue-specificity of lncRNAs (Djebali et al., 2012; Cabili et al., 2011; Ward et al., 2015; Li et al., 2015; Hon et al., 2017). The tissue specificity of age-lncRNAs most likely reflects this baseline tissue specificity, rather than suggest tissue-specificity of the ageing process itself.

However, protein coding genes co-expressed with the age-lncRNAs in different tissues were shown to overlap extensively, and the functional categories associated with the age-lncRNAs in different tissues showed overlap with each other. Functional categories associated with age-lncRNAs in different tissues also showed overlap with each other. Functional categories that were associated with the age-lncRNAs have also previously been linked to transcriptomic changes associated with ageing (Cellerino and Ori, 2017; Frenk and Houseley, 2018; de Magalhães et al., 2009). This suggests that while ageing-associated processes mostly are shared between tissues, the details of how the process occurs in each tissue may differ, at least at the level of lncRNA expression. Given the spatio-temporal specificity of lncRNA expression, lncRNAs most likely are fine-tuners, rather than master regulators, of the ageing-associated processes.

The up-regulation of lncRNAs showed more similarities across tissues as compared to down-regulation. The up-regulated age-lncRNAs were slightly less tissue-specific as compared to the down-regulated, and the genes co-expressed with the up-regulated age-lncRNAs showed more overlap and more shared functional categories as compared to the down-regulated. From population-based expression data, it is not possible to separate the changes that are contributing to ageing from those that are consequences of the ageing process. With lncRNAs, it is also possible that not all that is transcribed is functional (see Limitations). As the up-regulation of age-lncRNAs was found to be more coordinated across tissues, it is possible that among the up-regulated age-lncRNAs there are more changes that contribute to the ageing process, where as many of the down-regulated age-lncRNAs may represent transcriptomic noise and ageing-associated dysregulation of transcription.

### 4.4 Limitations

The present study suffers from some limitations. Number of ageing-associated genes and age-lncRNAs identified varied from tissue to tissue (Table 1 and Supplementary Table 1). A part of the variation is most likely explained by biological differences between the tissues, but technical artefacts also may have a role. Specifically, quality of RNA extraction (RIN value) varied between tissues (https://gtexportal.org/), and it has been shown to be associated with the expression profile (Ferreira et al., 2018). Therefore, true age-lncRNAs may have been excluded, which might exaggerate the number of tissue-specific age-lncRNAs.

As very few lncRNAs have been functionally characterised, we had to rely on co-expression analysis and known functions of protein coding genes to gain insight on the possible functions of age-lncRNAs. Possible lncRNA-specific functions are naturally lost in this type of analysis. Additionally, it is discussed in the field what proportion of lncRNAs actually are functional (Jandura and Krause, 2017), with estimates on the proportion of functional lncRNAs ranging from a few thousand to the majority of all described lncRNAs. Most likely some proportion of the identified age-lncRNAs lack function, and are expressed as by-products of purposeful transcription programs, which may be reflected in the co-expression analysis as well as the functional enrichment analysis. Therefore, the results of expression studies on lncRNAs need to be interpreted with caution, at least until a larger proportion of lncRNAs have been properly functionally characterised.

## 5 Conclusions

Here we report the ageing-associated changes in lncRNA expression in healthy human tissues. Our results show that age-lncRNAs are highly tissue-specific but are expressed with a common pool of protein coding genes and are associated with similar functional categories. lncRNA expression may contribute or reflect the tissue-specific fine-tuning of the ageing-associated process. Age-lncRNAs identified in the present study were associated with immune system processes as well as with signal transduction, transcription and translation, adding to the previous findings that these processes are affected with ageing at the transcriptomic level.

Study of lncRNAs related to the ageing process is hampered by the low number of functionally characterised lncRNAs. In order to gain a more nuanced picture of ageing-associated lncRNAs and to identify possible lncRNA-specific functions, a greater proportion of lncRNAs need to be functionally characterised. The present study and age-lncRNAs identified can be used as a resource in prioritising and selecting currently unknown lncRNAs for further functional studies.
