# Supplementary material for: Ageing-associated changes in the expression of lncRNAs in human tissues reflect a transcriptional modulation in ageing pathways
Source: Mech Ageing Dev. 2020 Jan;185:111177. doi: 10.1016/j.mad.2019.111177 (PMC6961210; doi:10.1016/j.mad.2019.111177)
Supplement: Supplementary file 1 [file mmc1.docx]

**List of supplementary material**

**Supplementary file 1.** Association of death circumstance with gene expression profiles of ageing.

**Supplementary table 1.** Lists of age-lncRNAs by tissue.

**Supplementary table 2.** Lists of protein coding genes co-expressed with up- and down-regulated age-lncRNAs.

**Supplementary table 3.** Overlap of co-expressed genes across tissues.

**Supplementary table 4.** Enrichment of GO terms for genes co-expressed with age-lncRNAs.

**Supplementary table 5.** Semantic similarity of enriched GO terms.

.
