# Supplementary material for: Ageing-associated changes in the expression of lncRNAs in human tissues reflect a transcriptional modulation in ageing pathways
Source: Mech Ageing Dev. 2020 Jan;185:111177. doi: 10.1016/j.mad.2019.111177 (PMC6961210; doi:10.1016/j.mad.2019.111177)
Supplement: Supplementary file 2 [file mmc2.docx]

**Supplementary file 1**

**Death circumstance is associated with age and gene expression levels**

Only samples from subjects who had died in a ventilator (Hardy scale 0) were included in the analyses in the accompanying manuscript. We have noted that the death circumstance, as measured with the Hardy scale, was associated with gene expression patterns and could potentially confound the identification of ageing-associated gene expression patterns.

Briefly, we performed a weighted correlation network analysis (WGCNA) (Zhang & Horvath, 2005; Langfelder & Horvath, 2008) for each tissue in GTEx database (dbGaP accession number phs000424.v7) to identify co-expressed gene modules and further identified the modules that are associated with ageing by correlating the expression level of the module eigengene with age. As shown in Figure S1, we identified several ageing associated modules, but almost all ageing-associated modules were also associated with death circumstance (Hardy scale score), and also showed a higher correlation coefficient and a lower p-value for Hardy scale as opposed to ageing.

We repeated the WGCNA for only the ventilator samples (Figure S2), and while we identified ageing-associated gene expression modules, there were fewer as compared to the analysis performed with all samples. This suggests that death circumstance has a significant effect on gene expression profiles, and more importantly, in the context of the current study, the effect is stronger than that of age. Previously it has been shown that the length of the post mortem interval (PMI) is associated with gene expression profiles (Ferreira et al., 2018) and that PMI is associated with death circumstance.

The percentage of individuals in each death circumstance category varies across ages (Table S1, (gtexportal.org)). Therefore, simply using death circumstance (Hardy scale) as a covariate in the regression model could potentially obscure true ageing-effects. To avoid this, we decided to use only samples form subjects with the same death circumstance, and chose the ventilator cases, as they were most numerous in the GTEx data.


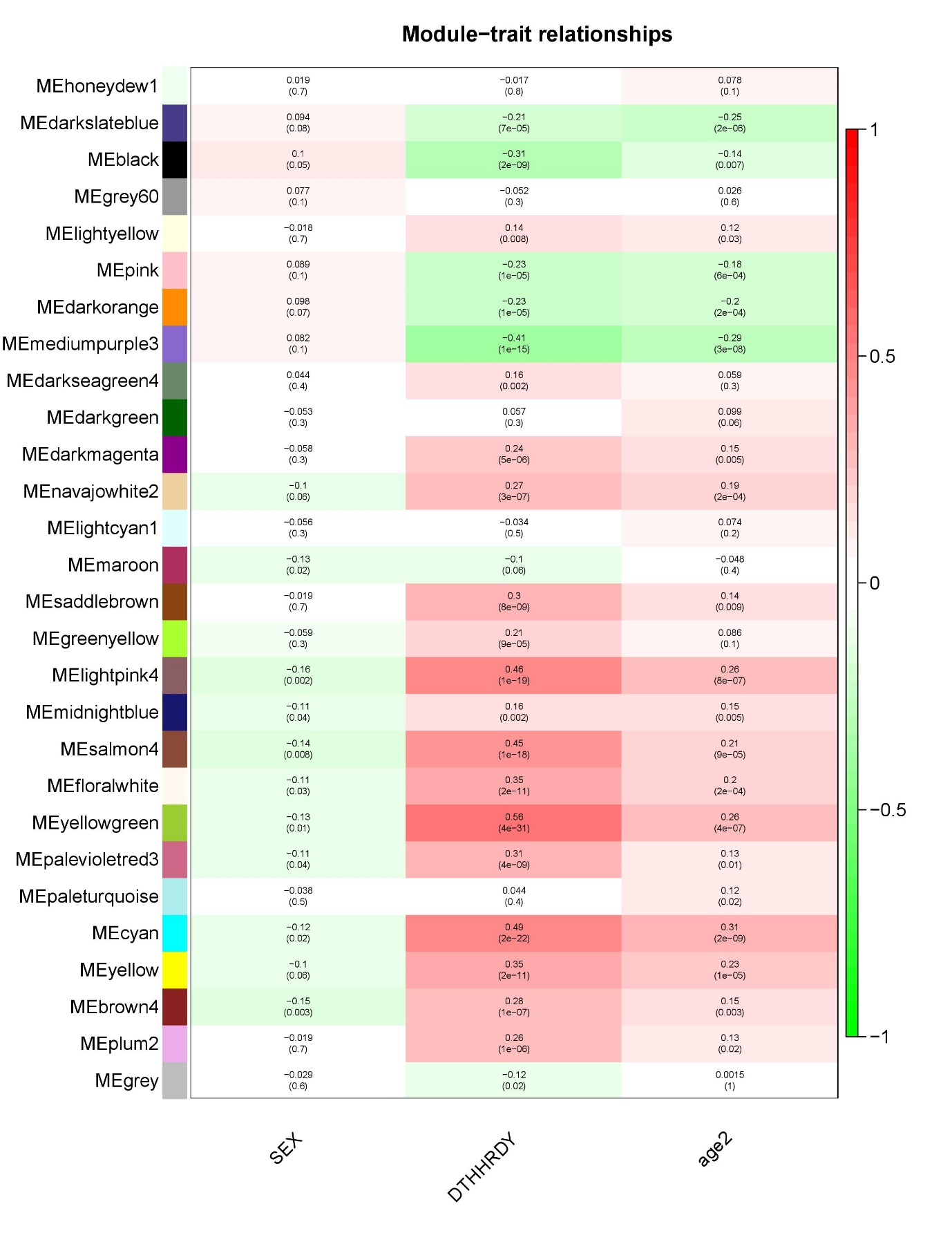
Figure S1. WGCNA with all samples in GTEx, correlation between module eigengenes (labelled with different colours on the left) and sex, death circumstance (Hardy scale, DTHHRDY) and age in adipose tissue. Presented are correlation coefficients and p-values. Majority of modules showing an association with age also show a stronger association with death circumstance.


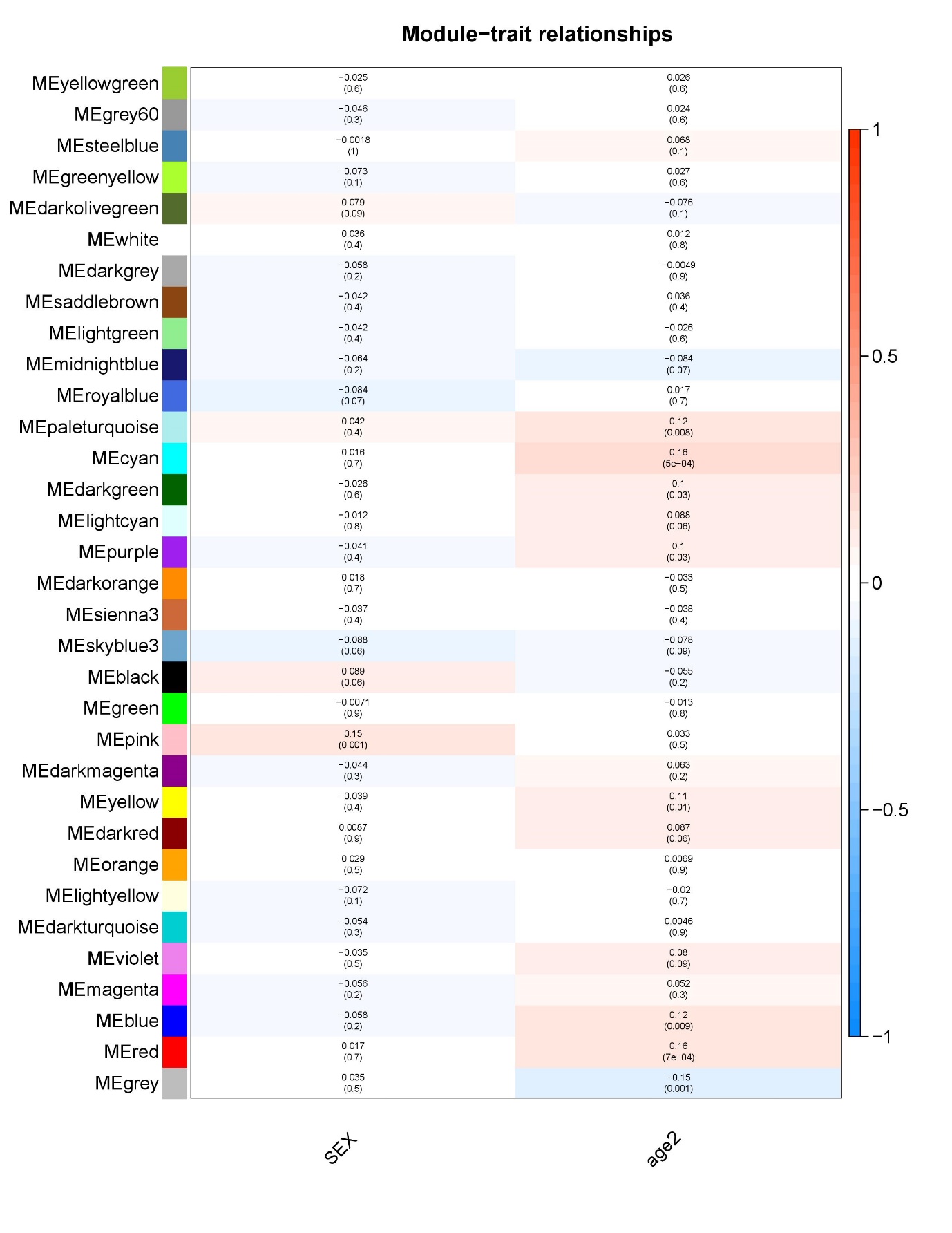
Figure S2*.* WGCNA with only ventilator samples in GTEx, correlation between module eigengenes (labelled with different colours on the left) and sex and age in adipose tissue. Please note the module labels are not comparable, i.e. cyan does not represent the same group of genes in both analyses (i.e. in Figures S1 and S2). There are fewer ageing-associated modules when only ventilator data is analysed, as compared to analysis performed with all samples available in GTEx.

|  |  | **Hardy scale (percentages)** | | | | |
| --- | --- | --- | --- | --- | --- | --- |
|  |  | **1** | **2** | **3** | **4** | **0** |
|  |  | Violent fast death | Natural fast death | Intermediate death (terminal phase 1-24 h) | Slow death | Ventilator |
| **Age group** | **20-29** | 13.79 | 3.45 | 1.72 | 0 | 81.03 |
|  | **30-39** | 7.84 | 7.84 | 0 | 5.88 | 78.43 |
|  | **40-49** | 4.1 | 13.93 | 2.46 | 5.74 | 73.77 |
|  | **50-59** | 3.27 | 30.2 | 4.49 | 8.98 | 53.06 |
|  | **60-69** | 1.27 | 36.44 | 9.32 | 20.34 | 32.63 |
|  | **70-79** | 4.76 | 33.33 | 9.52 | 23.81 | 28.57 |

Table S1. Percentage of subjects in each death circumstance category across ages.

References

Ferreira PG, Muñoz-Aguirre M, Reverter F, Sá Godinho CP, Sousa A, Amadoz A, Sodaei R, Hidalgo MR, Pervouchine D, Carbonell-Caballero J, Nurtdinov R, Breschi A, Amador R, Oliveira P, Çubuk C, Curado J, Aguet F, Oliveira C, Dopazo J, Sammeth M, Ardlie KG, Guigó R. The effects of death and post-mortem cold ischemia on human tissue transcriptomes. Nat Commun. 2018 Feb 13;9(1):490. doi: 10.1038/s41467-017-02772-x.

Langfelder P, Horvath S (2008) WGCNA: an R package for weighted correlation network analysis. BMC Bioinformatics 2008, 9:559

Zhang B and Horvath S (2005) A General Framework for Weighted Gene Co-Expression Network Analysis, Statistical Applications in Genetics and Molecular Biology: Vol. 4: No. 1, Article 17 PMID: 16646834
